# Genetic Substructure of Kuwaiti Population Reveals Migration History

**DOI:** 10.1371/journal.pone.0074913

**Published:** 2013-09-16

**Authors:** Osama Alsmadi, Gaurav Thareja, Fadi Alkayal, Ramakrishnan Rajagopalan, Sumi Elsa John, Prashantha Hebbar, Kazem Behbehani, Thangavel Alphonse Thanaraj

**Affiliations:** Dasman Diabetes Institute, Dasman, Kuwait; University of Florence, Italy

## Abstract

The State of Kuwait is characterized by settlers from Saudi Arabia, Iran, and other regions of the Arabian Peninsula. The settlements and subsequent admixtures have shaped the genetics of Kuwait. High prevalence of recessive disorders and metabolic syndromes (that increase risk of diabetes) is seen in the peninsula. Understanding the genetic structure of its population will aid studies designed to decipher the underlying causes of these disorders. In this study, we analyzed 572,366 SNP markers from 273 Kuwaiti natives genotyped using the illumina HumanOmniExpress BeadChip. Model-based clustering identified three genetic subgroups with different levels of admixture. A high level of concordance (Mantel test, p=0.0001 for 9999 repeats) was observed between the derived genetic clusters and the surname-based ancestries. Use of Human Genome Diversity Project (HGDP) data to understand admixtures in each group reveals the following: the first group (Kuwait P) is largely of West Asian ancestry, representing Persians with European admixture; the second group (Kuwait S) is predominantly of city-dwelling Saudi Arabian tribe ancestry, and the third group (Kuwait B) includes most of the tent-dwelling Bedouin surnames and is characterized by the presence of 17% African ancestry. Identity by Descent and Homozygosity analyses find Kuwait’s population to be heterogeneous (placed between populations that have large amount of ROH and the ones with low ROH) with Kuwait S as highly endogamous, and Kuwait B as diverse. Population differentiation F_ST_ estimates place Kuwait P near Asian populations, Kuwait S near Negev Bedouin tribes, and Kuwait B near the Mozabite population. F_ST_ distances between the groups are in the range of 0.005 to 0.008; distances of this magnitude are known to cause false positives in disease association studies. Results of analysis for genetic features such as linkage disequilibrium decay patterns conform to Kuwait’s geographical location at the nexus of Africa, Europe, and Asia.

## Introduction

The State of Kuwait is situated on the north-east of the Arabian Peninsula and at the northern end of the Persian Gulf. The Peninsula, which is at the nexus of Africa, Europe and Asia, has been implicated as part of early human migration route out of Africa [1,2] and of early inter-continental trade routes [3]. Historically, the population of Kuwait comprises early settlers originating from the tribes of neighboring Arabian and Persian countries (particularly from Saudi Arabia and Iran) and from the nomadic Arabs of the desert, living on the fringes of the Arabian Peninsula, called Bedouins [4,5]. Previous works with Kuwaiti population using Y-STR polymorphisms [6] have shown a pronounced similarity with Yemenis and Saudi Arabians. Mitochondrial DNA genetic variation studies [7], on individuals from three subgroups of the Kuwaiti population (of Saudi Arabian, Iranian or Bedouin ethnicity), have shown that (i) the three subpopulations of Kuwait are heterogeneous; (ii) the Kuwaiti population has a high frequency of those haplogroups (namely R0, J, and U) that are seen in other Arabian populations; and (iii) the maternal genetic structure of Kuwait resembles that of both Saudi Arabia and Iran. Similarly, works using autosomal and Y-STRs, and Y-SNPs with samples from nomadic Bedouins of Kuwait have shown evidence for genetic isolation and drift [8]. The population groups in Kuwait, like other states in the Peninsula, practice consanguineous marriage (with low frequency of intermarriage among the communities) [4]. The rate of consanguineous marriages can be as high as 54.3% with the average inbreeding coefficient at 0.02 [9]. These practices have led to the possibility of these groups living in isolation by community. A high level of genetic disorders, at least partly due to consanguineous marriage, is seen in Kuwait and other states of the Peninsula [10].

Kuwait, like other Gulf Cooperation Council (GCC) member states, has seen an unprecedented growth in its economy in the post-oil era. With the resultant increase in per capita income, a rise in the prevalence of lifestyle disorders (such as type 2 diabetes, obesity, and hypertension) [11,12,13] is also observed. Interactions between genetic background and changes in diet & lifestyle may accelerate the incidence of diabetes in the context of rapid nutrition transition [14]. The International Diabetes Federation, Atlas Fifth Edition [15] places Kuwait in the top 5 countries with highest prevalence of type 2 diabetes.

Recent advances in genome-wide association studies (GWAS) have provided a fairly good view of the contribution of common variants to complex traits [16] such as diabetes. However, while disease etiology is common between populations, risk variants can often be population-specific [17]. For example, although the rs7903146 and rs12255372 variants in TCF7L2 gene show strong associations with type 2 diabetes in most populations, they show weak or no association in Arab populations [18].

In this study, we examine whether the settlement history of Kuwait can be seen in the population substructures of Kuwait. We use genome-wide genotype data (derived from markers mapped in HumanOmniExpress BeadChip) from a pool of 273 unrelated samples. We compare the genetic structure of the Kuwaiti population with those of other continental populations. We also examine patterns of ROH (Runs of Homozygosity) and IBD (Identity By Descent) to illustrate population history and consanguinity. The study will pave the way for designing appropriate disease association studies in Kuwait and possibly in the entire Peninsula.

## Results

A data set of genotypes from 572,366 SNP markers in 273 samples was derived by genotyping 389 samples genome-wide and adopting extensive quality control. The ancestry distribution of the 273 samples in accordance with surname classification is found to be 100 (Persian), 109 (“city-dwelling” Saudi Arabian tribes), 33 (“tent-dwelling“ Bedouins), and 31 (unclassified).

### Population substructure in Kuwait

**Figure 1:**
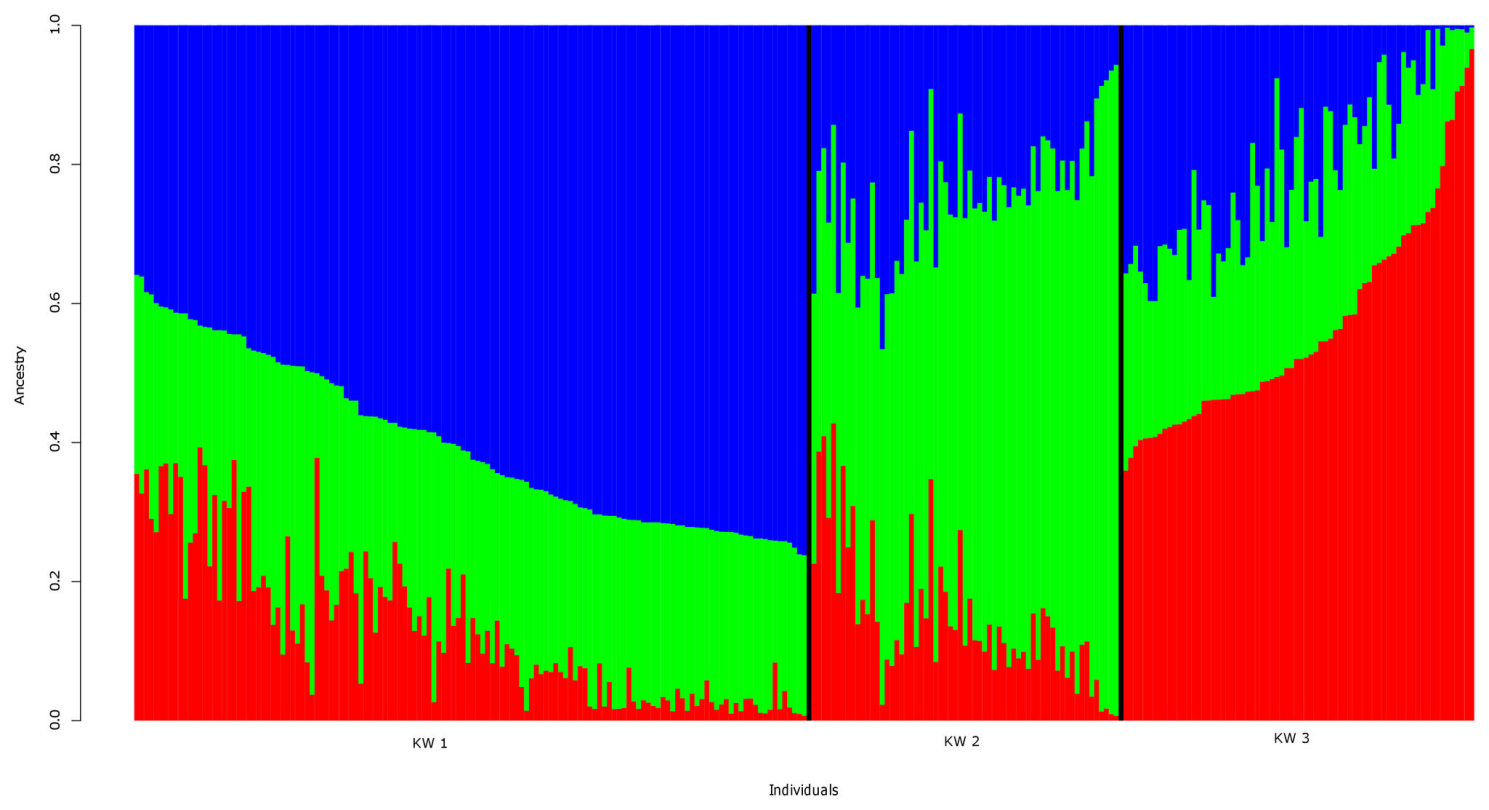
Ancestry estimates using STRUCTURE program for Kuwaiti samples. (Best model at K= 3). The three groups comprise 138, 63, and 72 individuals, respectively. For simplicity, we label the groups as Kuwait 1, Kuwait 2 and Kuwait 3 (from left to right). Mantel test correlation between pairs of all 4 separate runs (see Materials and Methods) is >0.99 with p-value = 1e-04 over 9999 replicates.

We used a model-based clustering approach, as implemented in STRUCTURE [19], to infer population structures in Kuwait. Different values ranging from 1 to 5 (based on previous history of Kuwait and the Peninsula) were assumed for K (number of subpopulations) in STRUCTURE calculations. K= 3 is selected, based on the values of mean est. LnP(Data) and other recommendations of the STRUCTURE software manual. Individuals were independently assigned to three affinity groups (called Kuwait 1 of 138 participants, Kuwait 2 of 63 participants and Kuwait 3 of 72 participants) using relative majority of likelihood assignment of individuals to subpopulations (Figure 1).

### Admixture of ancestries in the three Kuwaiti groups

**Figure 2:**
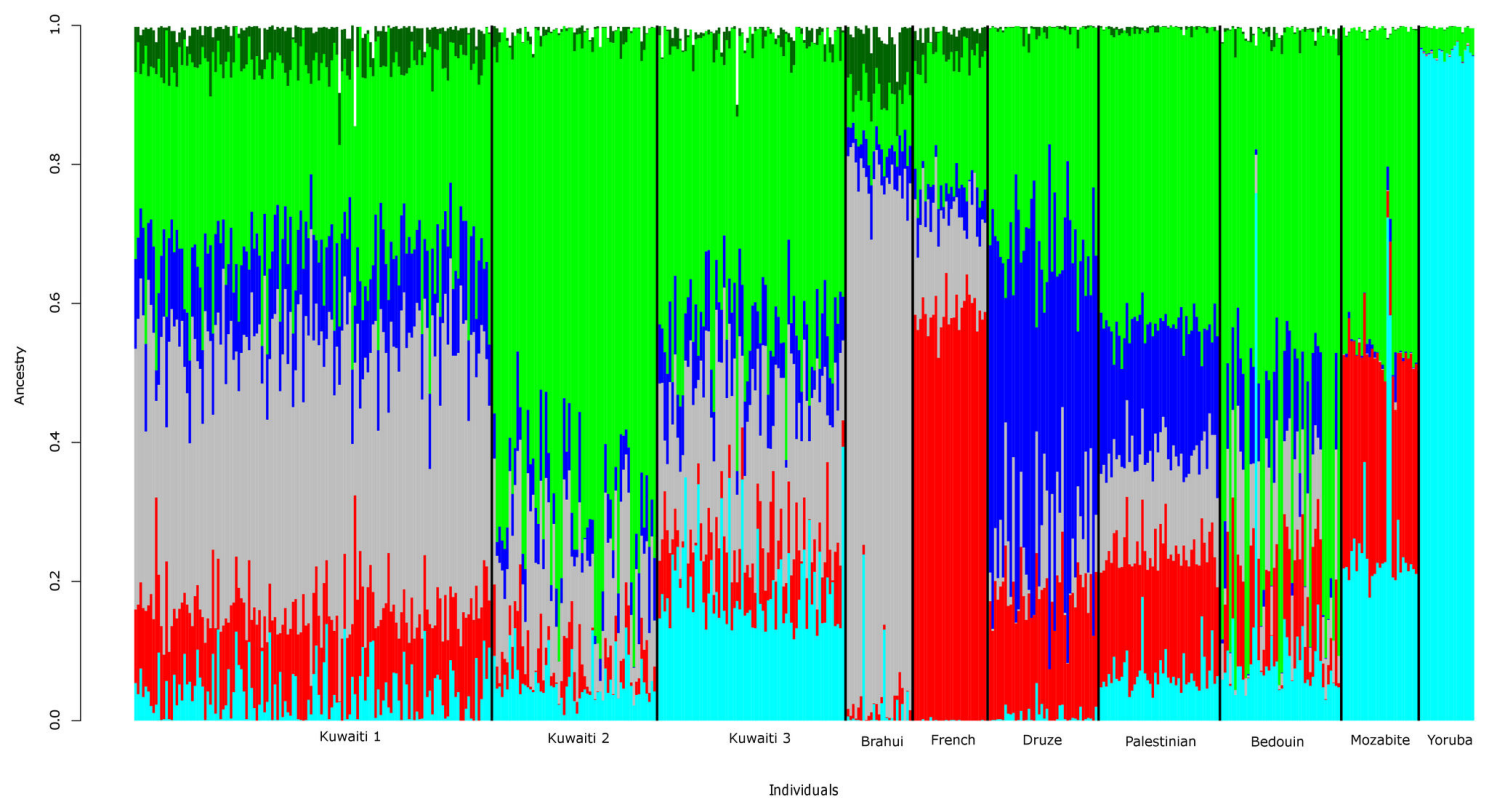
Ancestry composition of Kuwaiti groups using HGDP data. STRUCTURE results for the combined data set of three Kuwaiti groups and representative HGDP populations from West Asia (Brahui), Middle East (Bedouin, Druze and Palestinian), Mozabite (North Africa), Europe (French), and sub-Saharan Africa (Yoruba). Best Model for the combined data set is at K = 9. Structure results for the combined data set of three Kuwaiti groups and all of the HGDP populations are given in Figure S1. Red: French_Basque (Europe), Green: Bedouin (Arabs), Dark Green: Kalash (Asia), Cyan: Yoruba (sub-Saharan Africa), Blue: Druze (Persian) and Gray: Brahui as inferred from Figure S1. Black lines partition the groups.

Model-based ancestry estimation, using the combined data set of the three Kuwaiti groups and the populations from Human Genome Diversity Project (HGDP) [20], leads to the identification of six ancestral elements in the three Kuwaiti groups (see Figure 2 for results using combined data set with representative HGDP populations, and Figure S1 for results using combined data set with all of the HGDP populations). The Kuwaiti groups display admixture of 6 ancestral components that arise from the following HGDP populations: Negev Bedouin (from Israel, denoting Arabian ancestry), Yoruba (from sub-Saharan Africa), Brahui tribe (from Pakistan), Druze (from Israel), Kalash tribe (from Pakistan), and French Basque (from Europe). The observed extent of different ancestries in the three groups are as follows: West Asian (Brahui, Druze, and Kalash) ancestry is seen more in Kuwait 1 group (at 56%); European ancestry is seen more in Kuwait 1 group (at 12%); Arabian ancestry is seen more in Kuwait 2 group (at 69%) and in Kuwait 3 group (at 40%); and African ancestry is seen more in Kuwait 3 group at (17%). Thus, Kuwait 1 group is largely of Eurasian (Indo-Persian) origin; and Kuwait 2 and Kuwait 3 groups are largely of Arabian ancestry with Kuwait 3 group having a distinct African ancestry as well.

### Concordance between genetic clustering and surname-based ancestry of the participants

We found that 60% of the surnames in Kuwait 1 group are of Persian origin, and most of those with Persian surnames (83 of 100) are assigned to this group (Table 1). 81% of the surnames in Kuwait 2 group are of Saudi Arabian tribe origin and 36% of the surnames in Kuwait 3 group are of tent-dwelling Bedouin origin. Kuwait 3 group appears to be diverse, with a further 36% of the surnames as of Saudi Arabian tribe origin. However, most of the individuals with Bedouin surnames (26 of 33) are seen assigned to this group.

**Table 1:** Surname compositions of the derived genetic groups.

Genetic group	Persian surnames	Saudi Arabian tribe surnames	Tent-dwelling Bedouin surnames	Unclassified surnames	Count of individuals in each of the groups
Kuwait 1	83 (60.1%)	32 (23.19%)	3 (2.17%)	20 (14.49%)	138
Kuwait 2	4 (6.35%)	51 (80.95%)	4 (6.35%)	4 (6.35%)	63
Kuwait 3	13 (18.05%)	26 (36.11%)	26 (36.11%)	7 (9.72%)	72

Composition of surnames within each of the three Kuwaiti groups is presented. For example, 60.1% of the surnames included in Kuwait 1 group are of Persian origin. Mantel test comparing the genetic classification and surname origins indicates a very significant correlation (at p<0.0001 for 9999 repeats). The sizes of these groups do not necessarily represent the actual compositions in Kuwaiti population.

A Mantel test comparing the three subgroups and surname classifications indicated highly significant (p-value = 0.0001 for 9999 repeats) correlations across the three subpopulation groups with the frequencies of surname origins. The genetic clustering and surname-based determination of ancestries appear to be concordant. Based on historical lineage, we refer to Kuwait 1 group as Kuwait P (P for Persian origin), Kuwait 2 group as Kuwait S (S for Saudi Arabian tribe origin) and Kuwait 3 group as Kuwait B (B for Bedouin).

### *F*_*ST*_ distances between the three groups

F_ST_ statistics [21] are measures of population differentiation. F_ST_ distances between the three groups, calculated with adjustment for inbreeding, range from 0.005 to 0.008 (Kuwait P versus Kuwait S: 0.008 [st. dev: 0.000107]; Kuwait P versus Kuwait B: 0.005 [st. dev: 0.000079]; Kuwait S versus Kuwait B: 0.007 [st. dev: 0.000112]). Distances of this order of magnitude can cause false positives in disease association studies, as illustrated through simulated case-control studies with three substructures (with F_ST_ ranging from 0.0002 to 0.0009) in Han Chinese population [22].

### Inbreeding coefficients for the three groups

Enumeration of average inbreeding coefficient across individuals in each of the three groups (Table 2) indicates that Kuwait S has the highest average value at 0.04226 followed by Kuwait P at 0.025742 and Kuwait B at 0.00274 (Kruskal-Wallis test p-value < 0.001 & One-Way ANOVA p-value < 0.001). Kuwait B with such a low value for inbreeding coefficient and with 72% of individuals having negative inbreeding coefficients (Wilcoxon signed-rank test p-value = 0.03184) seems to exhibit negative assertive mating (i.e. more of inter-clan or intergroup marriages).

**Table 2:** Inbreeding coefficient distributions seen in the three Kuwaiti groups.

Range of Inbreeding coefficient	Percentage distribution of individuals		
	Kuwait P group	Kuwait S group	Kuwait B group
(-0.05 to 0.00)	33 (23.91%)	0 (0%)	52 (72.22%)
(0.00 to 0.05)	74 (53.62%)	43 (68.25%)	12 (16.67%)
(0.05 to 0.01)	24 (17.39%)	12 (19.05%)	7 (9.72%)
(0.01 to 0.15)	5 (3.62%)	7 (11.11%)	0 (0%)
(0.15 to 0.20)	2 (1.45%)	1 (1.59%)	1 (1.39%)
Total count of individuals in each group	138	63	72

### Trends in Identity by Descent and Homozygosity in Kuwaiti populations

**Figure 3:**
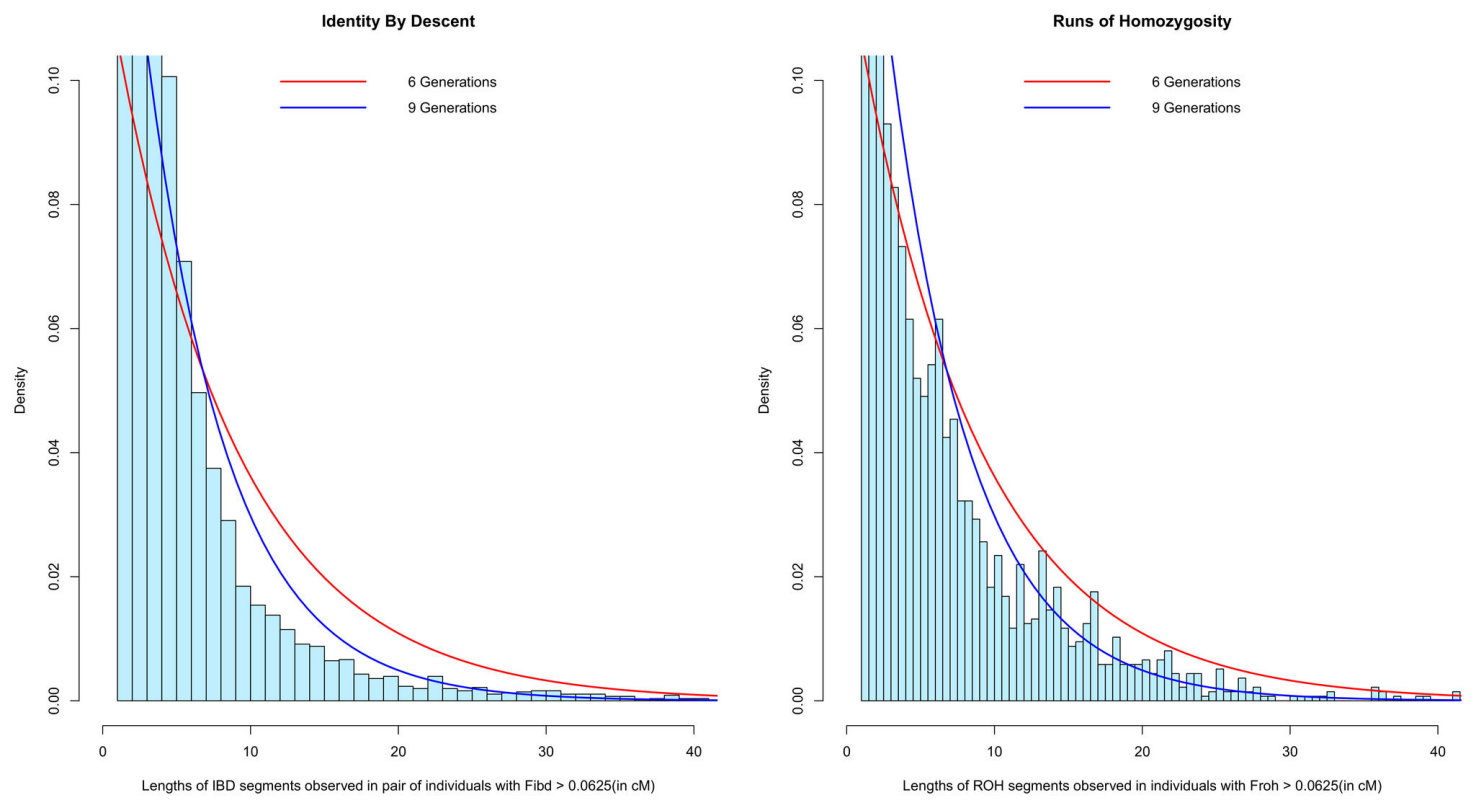
Length distributions of IBD and ROH segments shared among all of the Kuwaiti individuals (with F_roh_ > 0.0625 or F_ibd_ > 0.0625, as the case may be), and expected length distributions at different levels of inbreeding (6 or 9 generations since common ancestor). Considered are only those segments of length ≥ 1 cM. Assuming Haldanes’s recombination model, the length of segments should follow an exponential distribution with the mean as [1 / (2 × Number of generations since common ancestor)] in Morgans. The figure illustrates that the Kuwaiti population, as a whole, share a recent common ancestor within 6-9 generations. Figure S2 gives the distributions of IBD and ROH segments shared among individuals within each of the three Kuwaiti groups.

Identity By Descent (IBD) provides insights about shared common ancestry and sheds light on population history. We identify 102,060 segments (= 35.61% of the total segments) of length ≥ 2 cM shared among the 273 unrelated individuals; the average of the sum of all pairwise comparisons divided by the total number of segments shared per individual is seen to be 1.80 cM per individual with the longest segment measuring 84.87 cM. As high as 92.30% (= 252 individuals) of the cohort share at least one segment of length ≥ 10 cM with at least one another individual in the cohort. The total number of segments that are shared among all pairs of individuals follows an exponential distribution (Figure 3; Figure S2 presents the distribution considering separately each of the three Kuwaiti groups) indicating a recent common ancestor within 6 to 9 generations [23].

The distribution of total amount of IBD, total number of segments, and total number of segments versus total amount IBD (Figure S3) is distinctly different among the three Kuwaiti groups (One-Way ANOVA test on total amount of IBD: p-value = 5e-04; on total number of IBD Segments: p-value = 5.08e-08). Kuwait S has relatively high values for each of the above three parameters (Figure S3) and thus it can be inferred that this group is more inter-related. It is probably the case that Kuwait S has remained comparatively more endogamous than the other two groups. Though a significant amount of IBD can be seen shared (as inferred by shared long segments of length >= 10 cM) among individuals within each group, the number of segments shared between the groups is less (at 103, between Kuwait P and Kuwait S; at 186, between Kuwait P and Kuwait B; and at 195, between Kuwait S and Kuwait B).

Examination of Runs of Homozygosity (ROH) in individuals from the three Kuwaiti groups identifies a segment of length 41.33 cM and 2313 segments (= 5.67% of total segments) of length ≥ 2 cM. The total number of ROH segments follows an exponential distribution (similar to that seen in the case of IBD segments) showing shared ancestry within 6 to 9 generations (Figure 3; Figure S2 presents the distribution considering each of the three Kuwaiti groups). The distribution of total amount of ROH is not significantly different across the groups (One-Way ANOVA test on total ROH among the three groups: p-value = 0.129), whereas the total number of segments is significantly different between the groups (One-Way ANOVA test on total number of segments: p-value = 2.16e-09) (Figure S4). Interestingly, the Kuwait S group shows presence of both long and short segments implying both recent and ancient ancestry.

**Figure 4:**
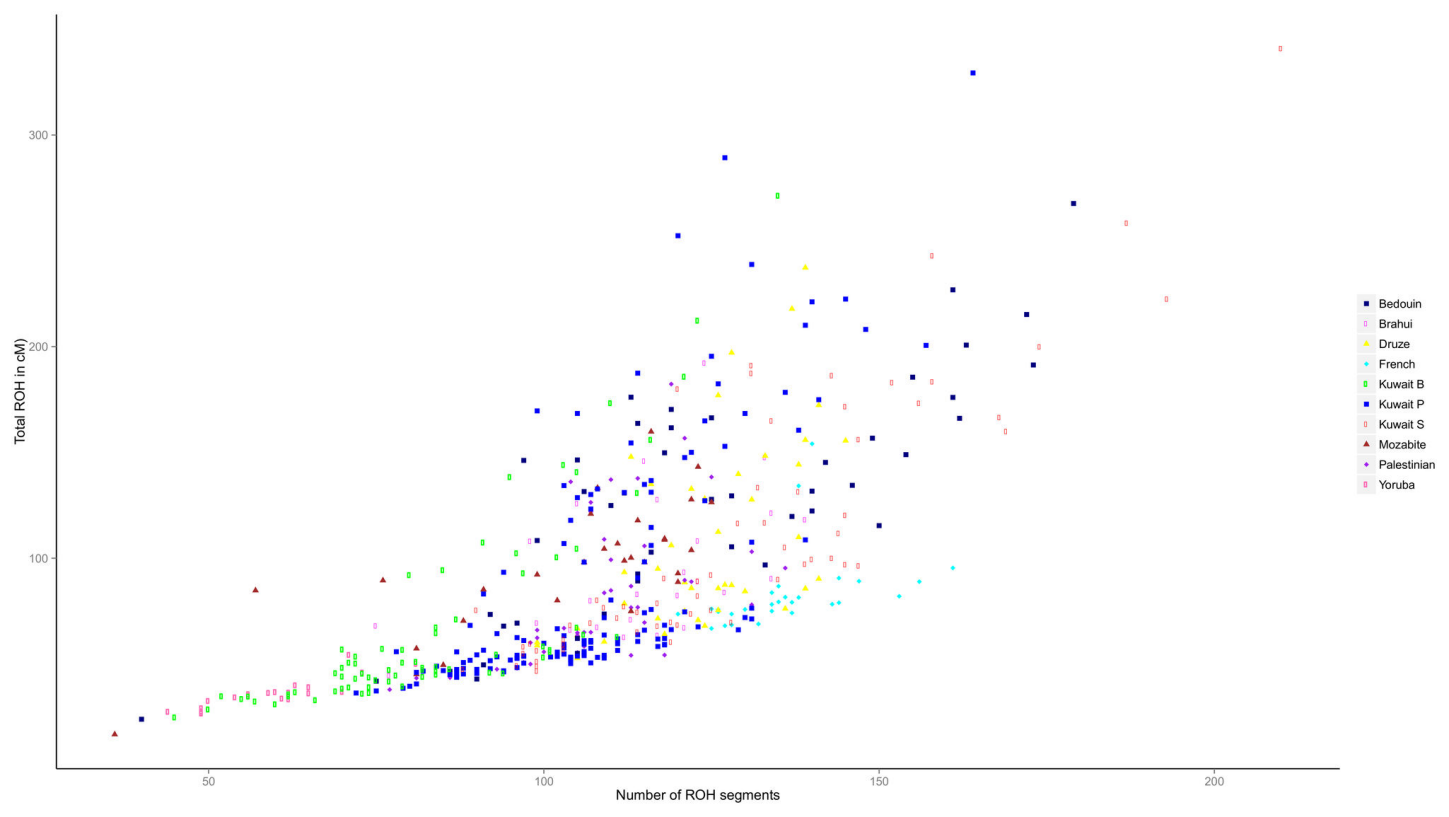
Plot of total amount of ROH versus total number of ROH segments in the three Kuwaiti groups and in representative HGDP populations. Kuwaiti groups exhibit a range of homozygosity showing characteristics of both consanguineous and non-consanguineous mating patterns. See Figure S5 for a representation for all of the Kuwait individuals in the background of all of the HGDP populations.

Examination of the patterns of ROH in individuals from the three Kuwaiti groups in the background of representative global populations from HGDP (Figure 4; see Figure S5 for representation in the background of all of the global populations from HGDP) indicates that the Kuwaiti population is placed between the groups that have large amount of ROH (showing isolation & high inbreeding as in Brazil and Columbia) and the ones that have low amount of ROH (as in sub-Saharan Africa and Europe).

### Relationship of Kuwaiti population with global populations

**Figure 5:**
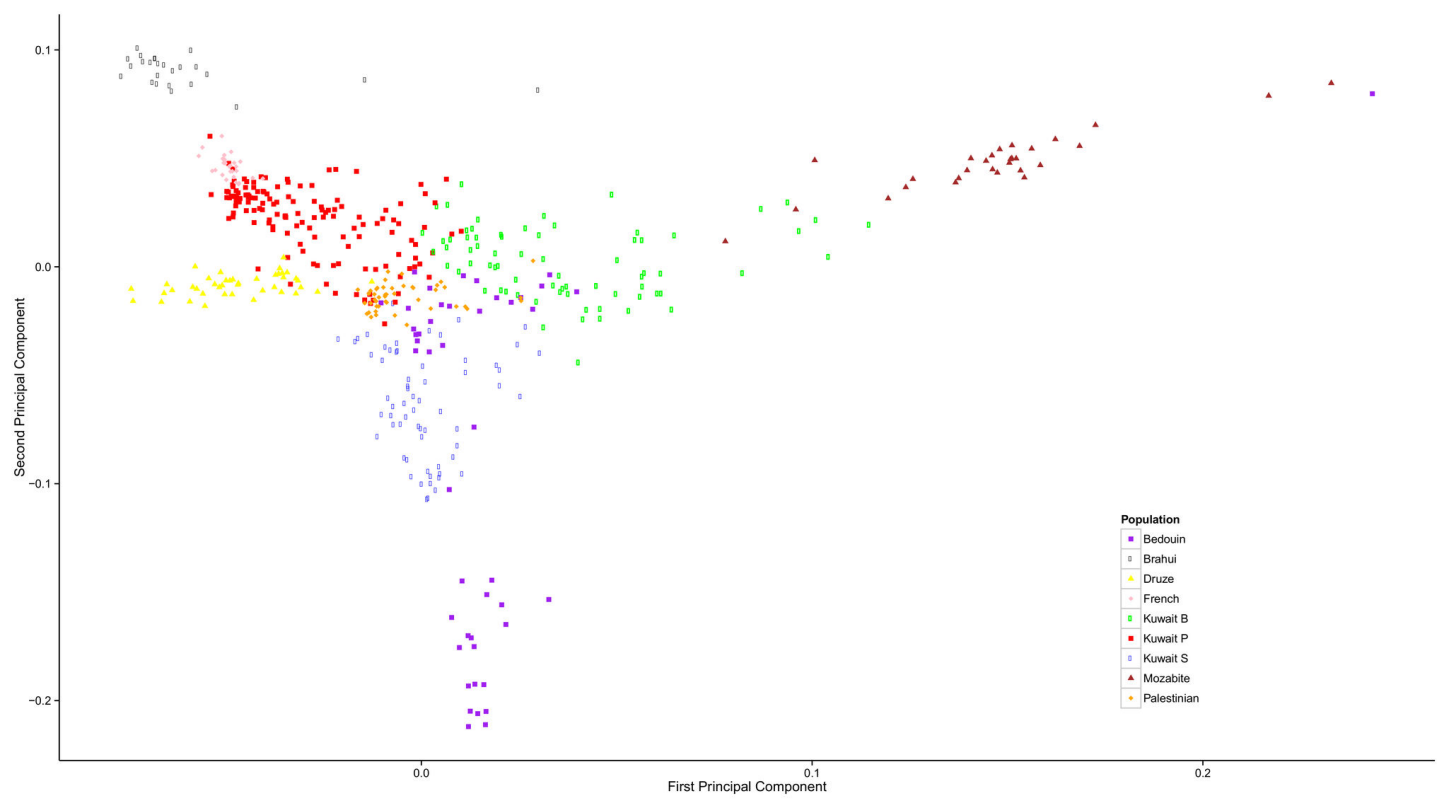
Scatter plot representing the first two principal components of merged data sets of the three Kuwaiti groups and representative HGDP populations The first principal component value ranges from 3.505-3.537 and the second Component value ranges from 2.688-2.710 in multiple iterations of PCA. See Figure S6 for similar plot derived by including Yoruba population.

**Figure 6:**
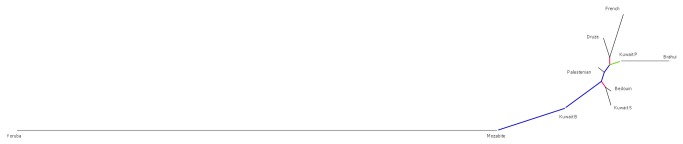
Plot (Phylogenetic tree) of pairwise F_ST_ distances for the three Kuwaiti groups with a set of representative HGDP populations that cover Europe, Asia, and Middle East. Bootstrap confidence – Red: 70% to 80%; Blue -80% to 90%; Green -90% to 100%.

Principal component analysis [24] of the genotype data from the merged data set of the three Kuwaiti groups and a subset of HGDP populations representing the different continents reveals clear distinction between the Kuwaiti groups and all other populations (Figure 5 and Figure S6). The three Kuwaiti groups are placed in the vicinity of (enveloped by) those from Middle East (Druze, Negev Bedouin and Palestinian), Indian subcontinent, Europe, and North Africa (Mozabite). Population differentiation F_ST_ estimates (see Figure 6 for Neighbor Joining tree) places Kuwait P group near to cluster of Asian populations, Kuwait S near the Negev Bedouin tribes, and Kuwait B near Mozabite population from Northern Africa. The F_ST_ estimates further confirm the placement of the Kuwaiti groups in concordance with geography and early migratory patterns in Eurasia.

### Linkage Disequilibrium decay rates for the three Kuwaiti groups

**Figure 7:**
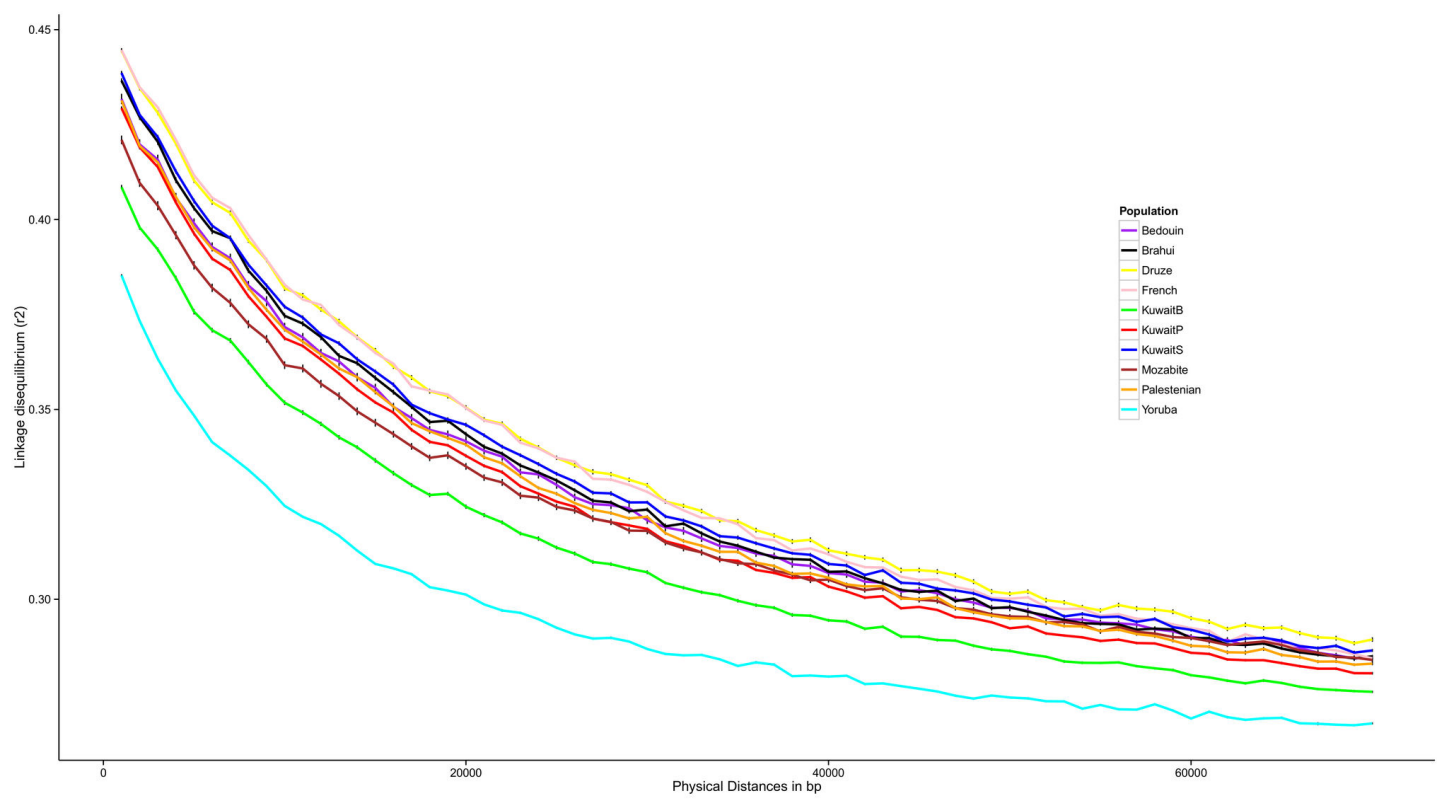
Linkage disequilibrium (LD) decay across the genomes of the Kuwaiti groups and other representative populations from the HGDP data set.

Figure 7 displays Linkage Disequilibrium (LD) decay rates (over increasing distances of base pairs) for the three Kuwaiti groups, and representative HGDP populations from Middle East (Druze and Palestinian), North Africa (Moabite), sub-Saharan Africa (Yoruba), West Asia (Brahui), and Europe (French Basque). The decay patterns exhibit similar rates across the populations but the conservation values are different at any given distance. Among the populations from Middle East, Kuwait B shows the lowest conservation values (only higher than the sub-Saharan African group) implying that the Kuwait B group is genetically more diverse.

## Discussion

The Kuwaiti native population is composed of three genetically distinct groups; Kuwait P is largely of Persian origin with admixture from European ancestries; Kuwait S is predominantly of city-dwelling Saudi Arabian tribe origin; and Kuwait B is a genetically diverse group characterized by inclusion of most of the tent-dwelling Bedouin participants and by presence of African ancestry (at 17%), a feature not seen with the other two groups.

The Structure plot (Figure 1) indicates that the three groups do not appear to be very homogeneous. The PCA plot (Figure 5) presents the Kuwait B as a cluster of thinly scattered points. The sparseness reflects the genetic diversity associated with Bedouins. Bedouin is a heterogeneous group ranging from camel nomads to more sedentary tribes who combined shepherding with scattered cultivation. Bedouins owe their origins to the deserts of Middle East and North Africa. Though almost all of the Bedouin surnames are included in Kuwait B, they constitute only 36% of the surnames in the group. The non-Bedouin surnames are seen spread over the entire cluster (Figure S7). Participants of non-Bedouin surnames in Kuwait B are reluctant to share further information on their ancestry and this reluctance can be attributed to socio-economic status of Bedouins in Kuwaiti society.

The population of Qatar, another region from the Arabian Peninsula, has also been partitioned into three genetic groups [25,26]. While the Persian group is seen in both the Kuwait and Qatar population, a predominantly African group is seen only in the Qatar population. The city-dwelling Saudi Arabian tribes and tent-dwelling Bedouins are seen as a single group in Qatar population. On the contrary, these are seen as two distinct subgroups in Kuwaiti population, the second group with an element of African ancestry at 17% (ranging from 11.7% to 39.4%). These two groups of Saudi Arabian tribes and Bedouin are distinctly different from one another and from the Persian group in many of the studied features such as inbreeding coefficients, LD decay rates, IBD and ROH patterns, and F_ST_ distances. In addition to the observed three groups, another tentative group of 10 members of predominantly Asian ancestry (that are currently removed as outliers and not used in the analysis - see Materials and Methods) can be extrapolated from the genotype results. Given that the population of the small State of Kuwait exhibits such heterogeneity, it is possible that the Arabian Peninsula is a mosaic of a number of subgroups with different ancestry compositions. Delineating this diversity is of importance to medical genetics in the region.

Results from ROH analysis suggest the existence of both modern outbred and consanguineous individuals, while the IBD analysis shows the existence of both recent and ancient shared ancestry between the three Kuwaiti groups. These three groups are seen critically placed between European and Indian populations in concordance with geography. Such an observation has also been made with Qatari populations from the Peninsula [25,26].

F_ST_ distances between the delineated three groups range from 0.005 to 0.008; distances of this magnitude can cause false positives in disease association studies [22]. Previous simulated genome-wide association studies have shown an increase in genome-wide χ^2^ inflation factor [27,28,29], an indicator of inflation in false-positive rates due to the presence of population substructures (or admixtures). Failure to take into account population stratification leads to an increase in the number of false positives and to a reduction in power of the study [22,30]. Upon performing simulated GWAS by randomly sampling case and controls between different pairs of subgroups in Kuwaiti population, we observe a similar increase in the Genome-wide χ^2^ inflation factor and decrease in power (data not shown). Future studies, that will lay emphasis on large-scale sampling of the population with collating exhaustive historical information and performing stringent genetics studies, are required to fully understand how the history of the settlements influenced the distribution of genetic variation in Kuwait.

In conclusion, we are able to break the Kuwait cohort down into distinct genetic groups that fall firmly into declared ancestral/tribal backgrounds. The population structure of Kuwait is heterogeneous but structured. Studies of disease association and risk allele identification need to consider these structures in the study designs. It will be interesting to next determine the genetic (recessive) disease load borne by these different groups. ROH analysis depicts the Kuwait S group as more endogamous than the other groups; this observation may begin to inform public health practice to target those populations for genetic testing with highest levels of ROH.

## Materials and Methods

### Ethics Statement

The study has been approved by the institutional ethics committee at Dasman Diabetes Institute, Kuwait. Written informed consent has been obtained from participants giving blood samples for the study. Data was analyzed anonymously.

### Participant recruitment

389 Kuwait natives were randomly recruited under protocols approved by the scientific and ethics advisory boards at our Institute. The participants were recruited through the following means: (i) from visitors to Open Day Events at our institute (ii) from visitors to our campaigns at malls, primary health centers and blood banks in each of the five governorates of Kuwait (thus maximizing the possibility to enroll people of different ancestries), and (iii) from visitors to our campaigns at Kuwait University, where students are enrolled from all the governorates. At the time of participant recruitment, the nationality was confirmed; since natives in Kuwait are sensitive to discussions on ancestral background, data on ancestry was not solicited in the first instance. Data on age, gender, lifestyle profile such as smoking, illness (e.g. diabetes and cardiovascular complications), and medication are recorded; further, vital signs such as height, weight, blood pressure readings and blood glucose levels are recorded.

### Sample collection and genotyping

Blood samples were collected in EDTA 4ml tubes. Gentra Puregene® kit (Qiagen, Valencia, CA, USA) was used to extract DNA as per manufacturer’s protocols. DNA was quantified, with a requirement that the A260/A280 ratio is in the range of 1.8–2.1, using both Quant-iT™ PicoGreen® dsDNA Assay Kit (Life Technologies, NY, USA) and Epoch Microplate Spectrophotometer. Frozen DNA stocks were diluted to a working solution of concentration at 50 ng/µl as recommended by Illumina (Illumina, CA, USA). Genotyping assay includes whole genome amplification, fragmentation, hybridization, staining and imaging of HumanOmniExpress BeadChips using the Illumina iSCAN system.

### Quality control on samples and markers

Genotyping was performed in 16 batches. In each of the batches, samples from Kuwaiti natives were genotyped along with samples from other ethnicities as part of a large study. Extensive quality control was performed to minimize effects due to batch composition. We performed genotype calling in 3 different ways (as below) to ascertain the quality of genotype calls.

#### Method 1

Genotypes were called for every batch, irrespective of the ethnic composition of the batch, using default parameters and cluster coordinates for the GenCall algorithm provided by Illumina GenomeStudio software. Samples with call rates < 97% were removed. Genotype calls of Kuwaiti natives from each batch were pooled. 

#### Method 2

Raw intensity data of all samples from the 16 batches were pooled and genotypes were called using GenCall algorithm. Samples with call rates < 97% were removed. A series of quality metric thresholds were applied (GenomeStudio software) to derive a data set of high quality SNPs. For every retained SNP, the following criteria were ascertained: intensity (AB R mean > 0.25); cluster separation (Cluster Sep ≥ 0.3); heterozygote clusters should not be too close to homozygotes (AB T Mean  > 0.2 and < 0.8); and heterozygotes should not be too excessive or too few (Het Excess > -0.3 and < 0.2). Finally, only those SNPs with a call rate of ≥ 98% were retained.

#### Method 3

Raw intensity data of only the Kuwaiti samples from all the batches were pooled together. Unsupervised clustering of markers was performed and genotypes were called using GenCall algorithm. Quality control steps, similar to Method 2, were carried out to derive high quality Kuwait genotype data.

A concordance rate of 99.9% was seen in genotype calls from the above three methods. These concordant SNPs were scrutinized further. We removed those markers that do not map to any genome regions (as per Human Genome Build 37.3), that map to non-autosomal chromosomes, that are multi-allelic or of A/T and G/C type, that show low minor allele frequency values (≤ 0.05) or that are not in Hardy-Weinberg Equilibrium (P < 1e^-3^). We also removed samples with call rate < 0.97 (total of 9 samples removed), or gender mismatch (17 samples removed), or inconsistent ancestry (23 samples removed). By “inconsistent ancestry”, we mean that these participants have very high (≥ 50%) Asian ancestry. These participants were generally seen to have Asian mothers. Samples that are related up to the levels of second cousins were identified and only one of such related samples was retained (67 samples removed).

10 samples from multiple batches were replicated by laying them in a new batch that is predominantly of Kuwaiti samples. A concordance of 99.9% was observed in the genotype calls from the two experiments.

All quality control steps were carried out using SNP Variation Suite 7.7 (SVS) (Golden Helix Inc http://www.goldenhelix.com/).

### Final set of markers and samples after quality control steps

The extensive quality control steps led to a final data set of 572,366 SNP markers and 273 samples. Distributions of age and other characteristics of these participants are as below: Gender: (M = 126, F = 147); age: (10-19 years = 4; 20-39 years = 87; 40-59 = 111; 60-79 years = 69; 80-90 years = 2); body mass index (< 18.5 = 2; 18.5 to 24.9 = 40; 25 to 29.9 = 77; 30 to 34.9 = 65; 35 to 39.9 = 34; ≥ 40 = 52); diabetes status (diabetic = 140, non-diabetic = 130); and cardiovascular complications (yes = 27, no = 245).

### Surname lineage classifications to decipher the ancestry of participants

The naming convention in Kuwait is, in general, as below: the first name is the personal name. The second name is the father’s personal name. The third and fourth names are the grandfather’s personal name and a name that denotes the family lineage. Women do not take the husband’s name upon marriage.

The methodology to assign ancestry through examining surnames considers the first, middle, and last names that together point to the name and ancestry of founder tribe. A team of three researchers from the region was formed to examine the surnames. Ethnicity assignment is obvious when the surname refers to an established tribe name. In other instances, further considerations were applied: a) using the websites that are populated with information on surnames from multiple resources on genealogy, b) consulting with natives who are familiar with tribe names, and c) discussing with the individual participants to clarify on their ancestral affiliations. Consistent calling of ancestry was performed to annotate the surnames as either Persian, or Saudi Arabian Tribe, or Bedouin or Unclassified. An individual is denoted as Persian if the surname can be traced back to a tribe from Iran; as Saudi Arabian tribe if the surname can be traced back to the tribes that inhabited the major cities of ancient (i.e. at least as back as the period of Prophet Muhammad) Saudi Arabia; as Bedouin if the surname can be traced back to the nomads that inhabited the deserts of Middle East and North Africa (particularly of Arabia, Jordan, Syria, and parts of the Sahara) and that moved around to follow seasonal grazing and water resources. The Bedouin is a heterogeneous group ranging from camel nomads to more sedentary tribes who combined shepherding with scattered cultivation. The Bedouins can be more generically referred as tent dwellers as opposed to descendants of Arabian ancient tribes that formed leadership class and are land owners.

### Derivation of substructures in Kuwaiti population

Linkage Disequilibrium (LD) pruning was performed on all Kuwaiti samples using the Composite Haplotype Method (CHM) (from SVS) with a window size of 50, a window increment of 5, and an r^2^ threshold of 0.1. This trimmed data containing 37,395 SNP markers was used in multiple runs of the STRUCTURE program [19] to cluster individuals into affinity groups. Four replicate runs using a burn-in period of 10,000 iterations followed by 100,000 iterations were performed from which admixture estimates were obtained. All runs were based on admixture model, in which each individual is assumed to have ancestry in multiple genetic clusters; the model further assumes that allele frequencies in each population are independent and are drawn from a distribution that is specified by a parameter called λ. The value for λ was set to 1. The program was executed by letting K (number of subpopulations) to assume values from 1 to 5. For all the five derived models, summary reports and CLUMPP input files were generated using STRUCTURE HARVESTER tool [31]. The value with highest mean est. LnP (Data) was used to choose the optimal value for K as 3 (Table S1). In order to confirm that the runs have converged after 10,000 iterations, we examined the plot of Alpha values (Figure S8). The multiple iterations for the value of K=3 were merged using CLUMPP [32] (with the run time parameters as individual data in the file, 4 runs and Full Search). Further, the Mantel correlation coefficient was calculated across multiple runs to identify consistency in admixture proportions for each of the individuals in the cohort across multiple runs. The samples were then classified into 3 groups using the method of relative majority of likelihood assignment of individuals to subpopulations.

### Genotype data from global populations

Genotype data on 52 populations from Human Genome Diversity Project (HGDP, http://hagsc.org/hgdp/files.html) were pooled together upon which quality control steps for SNP and sample call rates were carried out. A published list of unrelated individuals [33] in HGDP data was used to remove relatedness. All the above populations were then merged with the Kuwaiti population using SNP Variation Suite (SVS), and where necessary, strand flipping was performed using PLINK version 1.07 [34].

### Delineating the ancestry elements in Kuwaiti groups

Merged data set of Kuwaiti groups and all of the HGDP populations was LD pruned at r^2^ threshold of 0.1 leaving 39,589 markers using similar method as with Kuwaiti population. This data set was used to evaluate genetic admixture in the identified Kuwaiti groups using STRUCTURE program. Three replicate runs using a burn-in period of 10,000 iterations followed by 10,000 iterations were performed from which admixture estimates were obtained. All runs were based on admixture model, in which each individual is assumed to have ancestry in multiple genetic clusters; the model further assumes that allele frequencies in each population are independent and are drawn from a distribution that is specified by a parameter called λ. The value for λ was set to 1. The program was executed by letting K (number of subpopulations) to assume values from 1 to 15. For all the derived 15 models, summary reports were generated using STRUCTURE HARVESTER tool. The value with highest mean est. LnP (Data) was used to choose the optimal value for K as 9. The run with highest mean value of ln likelihood for the chosen K was used to derive the STRUCTURE plots (Figure 2 and Figure S1). Of the 9 ancestries thus identified (individually color-coded in Figure S1), only 6 ancestries (individually color-coded in Figure 2) are seen to contribute to admixture in Kuwaiti population.

### F_ST_ Distance Calculations

F_ST_ distances between the Kuwaiti groups were calculated using SmartPCA and were adjusted for inbreeding [35] using the entire data set of 572,365 markers. The error rates were calculated using 553 iterations of ‘moving block jackknife’ procedure.

### Inbreeding Coefficient Calculations

Inbreeding coefficient was calculated using SVS on merged Kuwaiti groups. In any given population, there is a certain level of homozygosity; inbreeding coefficient, F is simply an estimate of the increase from that initial level of homozygosity as a result of recent inbreeding. Rather than calculating the initial level of homozygosity per group, they are estimated over the entire Kuwaiti groups.

### Identity by Descent (IBD) Calculations

The entire data set of samples from all the three Kuwaiti groups was used to calculate IBD segments using the fastIBD program [36]. Conversion of PLINK format to BEAGLE format was done using the germline software [37]. The SNPs that lie in centromere regions (as per data downloaded from UCSC website [38]) were removed before performing fastIBD. We performed 10 rounds of fastIBD using random seeds on unphased Kuwait data set. The 10 iterations were merged using the fibd.py script available on BEAGLE website with the criteria that a confidence value of < 1e-10 is required to report an IBD segment. For all SNPs, the physical positions were converted to genetic marker positions using genetic marker map downloaded from Illumina website.

### Run of Homozygosity (ROH) Calculations

SVS was used to calculate ROH segments. For analyzing ROH in the three Kuwaiti groups, the entire data set of 572,365 makers was used with the following run parameters: minimum distance of 500 kb with a minimum of 25 SNPs, allowing a run to contain up to 1 heterozygote and up to 5 missing genotypes, and allowing a maximum gap of 100 kb between SNPs in a run. For all the SNPs, the physical positions were converted to genetic marker positions using genetic marker map downloaded from Illumina website.

Merged data set of Kuwaiti groups and representative or all of the HGDP populations was used, with similar settings as above, to calculate ROH segments that are used to derive Figure 4 or Figure S5, as the case may be.

### Principal Component Analysis

Merged data set of Kuwaiti groups and representative groups of HGDP populations from different continents was created. The representative population groups are Brahui (West Asia); French (Europe); Druze, Palestinian, Bedouin (Arabian Peninsula); Mozabite (North Africa); and Yoruba (sub-Saharan Africa). The data set of 240,461 markers was used to estimate principal components using SmartPCA [39]. To ensure that the PCA is not distorted by different population sizes, we implemented a ‘drop one in’ procedure [40]. Specifically, PCA analysis was performed for each individual from a Kuwaiti group along with all other HGDP samples. The resultant PC coordinates for the first two components from each Kuwaiti sample were then plotted together with the average PC coordinates from each HGDP sample across all runs.

### Derivation of Phylogenetic Tree using F_ST_ distances

The merged data set of Kuwaiti groups and representative HGDP populations was used to derive phylogenetic tree. Five distinct samples from each of the 10 populations were randomly selected to calculate F_ST_ distance using SmartPCA with adjustment for inbreeding. This selection procedure was repeated 1000 times and a consensus tree was generated using neighbor joining method as implemented in PHYLIP package [41]. The tree was visualized using Dendroscope package [42].

### Linkage Disequilibrium Decay Calculations

Pairwise r^2^ LD values were calculated for all SNPs in a distance of 1 Mb using PLINK. For each population (the three Kuwaiti groups and representative HGDP populations) 5 samples were randomly drawn without replacements and the LD values were calculated independently. The above step was repeated 50 times and error bars at 95% confidence level were calculated. The LD decay plot (Figure 7) was derived by defining a bin size of 1 kb (including the lower endpoint in the bin) for SNP pairs. For each population, starting at 500bp, the mean r^2^ value was calculated for 1kb bin. As an example, the value plotted at 1kb is the mean of r^2^ for all SNP pairs with physical distance in (950, 1050) [43].

### Data Sharing

Illumina OmniExpress SNP genotype data of Kuwaiti samples in PLINK Format is provided in Table S2. The LD pruned data contains 37,395 SNP markers from 273 samples.

## Supporting Information

Figure S1 **STRUCTURE results for the combined data set of the three Kuwaiti groups and all of the HGDP populations.** Best Model for the combined data set is at K = 9. Structure results for the combined data set of three Kuwaiti groups and representative HGDP populations are given in Figure 2. Red: French_Basque (Europe), Green: Bedouin (Arabs), Dark Green: Kalash (Asia), Cyan: Biaka_Pygmies and Yoruba (sub-Saharan Africa), Blue: Druze (Persian), Purple: Han Chinese, Pink: America, Yellow: Oceania and Gray: Brahui. Black lines partition the groups.
(TIFF)

Figure S2 **Length distributions of IBD and ROH segments shared among individuals within each of the three Kuwaiti groups (with F_roh_ > 0.0625 or F_ibd_ > 0.0625, as the case may be), and expected length distributions at different levels of inbreeding (4 or 6 generations since common ancestor).** Considered are only those segments of length ≥ 1 cM. Assuming Haldanes’s recombination model, the length of segments should follow an exponential distribution with the mean as [1 / (2 × Number of generations since common ancestor)] in Morgans. Figure 3 gives the distributions of IBD and ROH segments shared among all of the Kuwaiti individuals. Differences in sample sizes may account for the observation of 4-6 generations while considering each of the three Kuwaiti groups as opposed to 6-9 generations while considering the data set of all the three groups put together.
(TIF)

Figure S3 **Distributions of total amount of IBD, total number of IBD segments, and total amount of IBD versus total number of IBD segments in each of the three Kuwaiti groups.**
(TIF)

Figure S4 **Distributions of total amount of ROH, total number of ROH segments, and total amount of ROH versus total number of ROH segments in each of the three Kuwaiti groups.**
(TIF)

Figure S5 **Plot of total amount of ROH versus total number of ROH segments between the Kuwaiti population and all of the HGDP populations.** See Figure 4 for a representation for all of the Kuwait individuals in the background of only the representative HGDP populations.
(TIF)

Figure S6 **Scatter plot representing the first two principal components of merged data sets of the three Kuwaiti groups and the representative HGDP populations.** **The** first principal component value ranges from 11.517-11.547 and the second component value ranges from 2.79-2.805 in multiple iterations of PCA. See Figure 5 for similar plot derived by excluding Yoruba population.
(TIF)

Figure S7 **The PCA plot (namely the Figure S6) reproduced with color-coding the Bedouin and non-Bedouin surnames from the Kuwait B group.** Individuals with non-Bedouin surnames, represented by Cyan color, are seen spread over the entire cluster of Kuwait B.
(TIF)

Figure S8 **Distribution of Alpha values for 4 runs of STRUCTURE at K = 3 to identify substructure in Kuwait.**
(TIF)

Table S1 **Distribution of mean est. LnP value across multiple runs of STRUCTURE used to choose the value of K = 3.**
(DOCX)

Table S2 **Illumina OmniExpress SNP genotype data of Kuwaiti population sample in PLINK Format.** The LD pruned data contains 37,395 SNP markers from 273 samples.
(ZIP)
