# Supplementary figures and images for: Genetic Substructure of Kuwaiti Population Reveals Migration History
Source: PLoS One. 2013 Sep 16;8(9):e74913. doi: 10.1371/journal.pone.0074913 (PMC3774671; doi:10.1371/journal.pone.0074913)

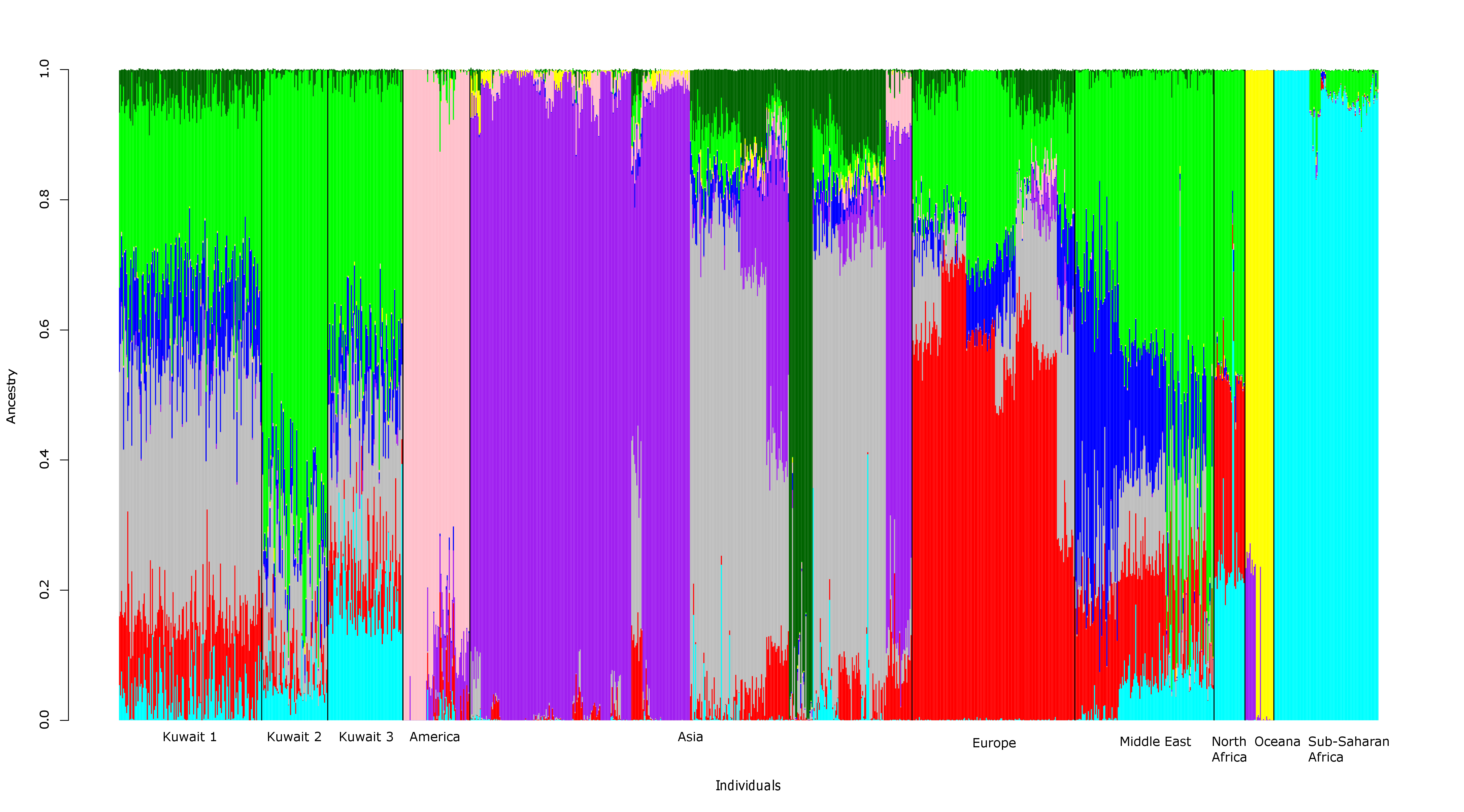

Supplement: Figure S1 — STRUCTURE results for the combined data set of the three Kuwaiti groups and all of the HGDP populations. Best Model for the combined data set is at K = 9. Structure results for the combined data set of three Kuwaiti groups and representative HGDP populations are given in Figure 2. Red: French_Basque (Europe), Green: Bedouin (Arabs), Dark Green: Kalash (Asia), Cyan: Biaka_Pygmies and Yoruba (sub-Saharan Africa), Blue: Druze (Persian), Purple: Han Chinese, Pink: America, Yellow: Oceania and Gray: Brahui. Black lines partition the groups. (TIFF) [file pone.0074913.s001.tiff]

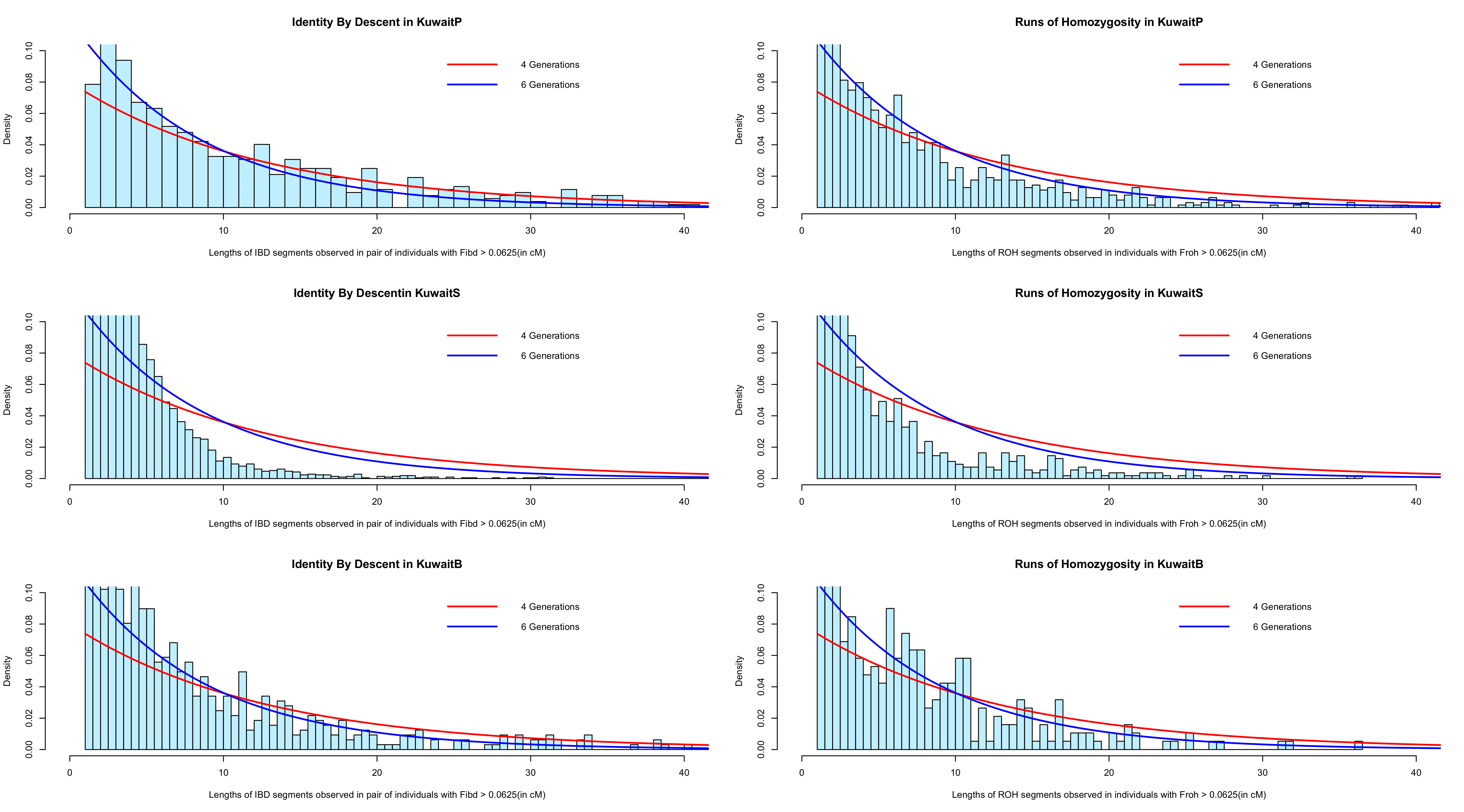

Supplement: Figure S2 — Length distributions of IBD and ROH segments shared among individuals within each of the three Kuwaiti groups (with Froh > 0.0625 or Fibd > 0.0625, as the case may be), and expected length distributions at different levels of inbreeding (4 or 6 generations since common ancestor). Considered are only those segments of length ≥ 1 cM. Assuming Haldanes’s recombination model, the length of segments should follow an exponential distribution with the mean as [1 / (2 × Number of generations since common ancestor)] in Morgans. Figure 3 gives the distributions of IBD and ROH segments shared among all of the Kuwaiti individuals. Differences in sample sizes may account for the observation of 4-6 generations while considering each of the three Kuwaiti groups as opposed to 6-9 generations while considering the data set of all the three groups put together. (TIF) [file pone.0074913.s002.tif]

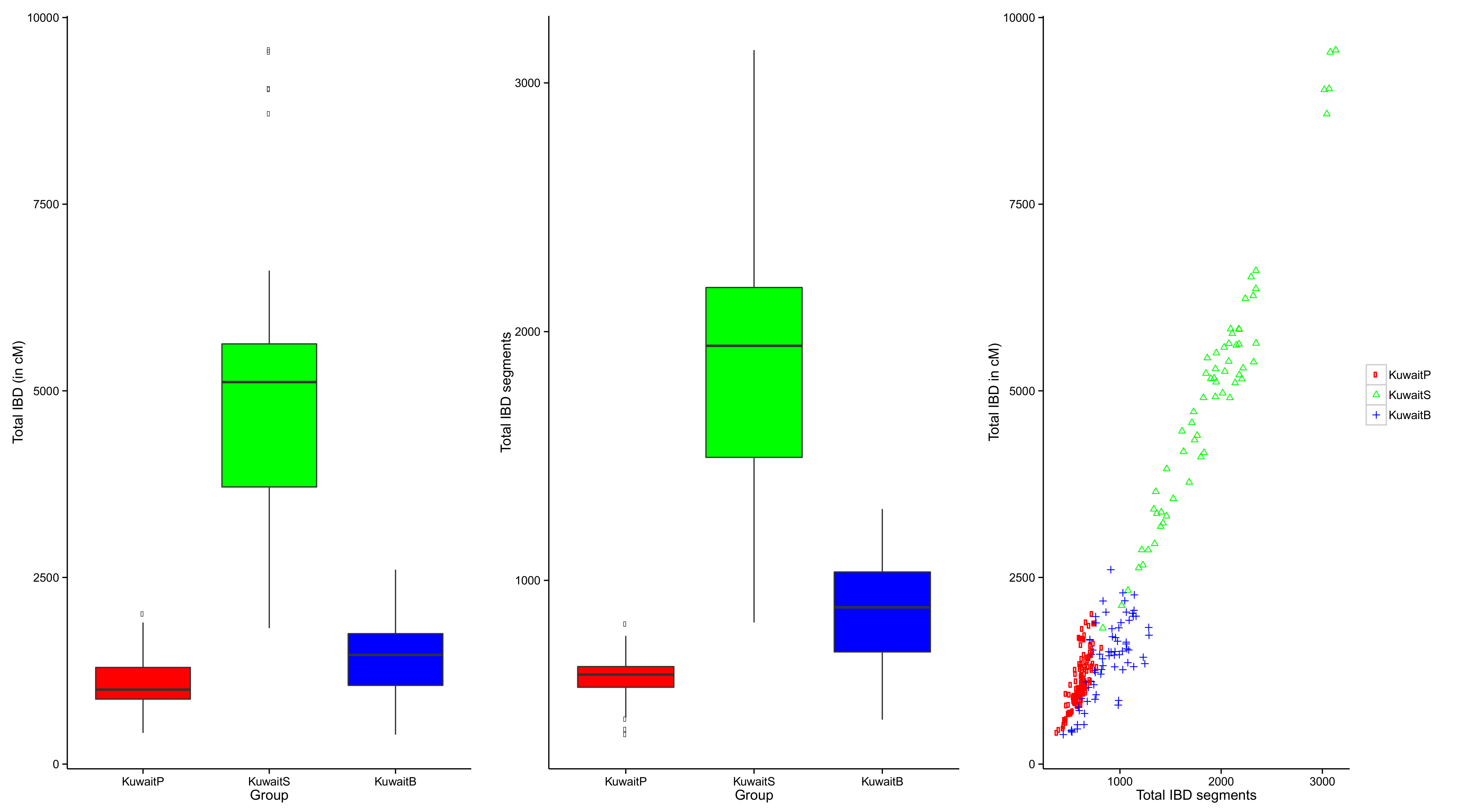

Supplement: Figure S3 — Distributions of total amount of IBD, total number of IBD segments, and total amount of IBD versus total number of IBD segments in each of the three Kuwaiti groups. (TIF) [file pone.0074913.s003.tif]

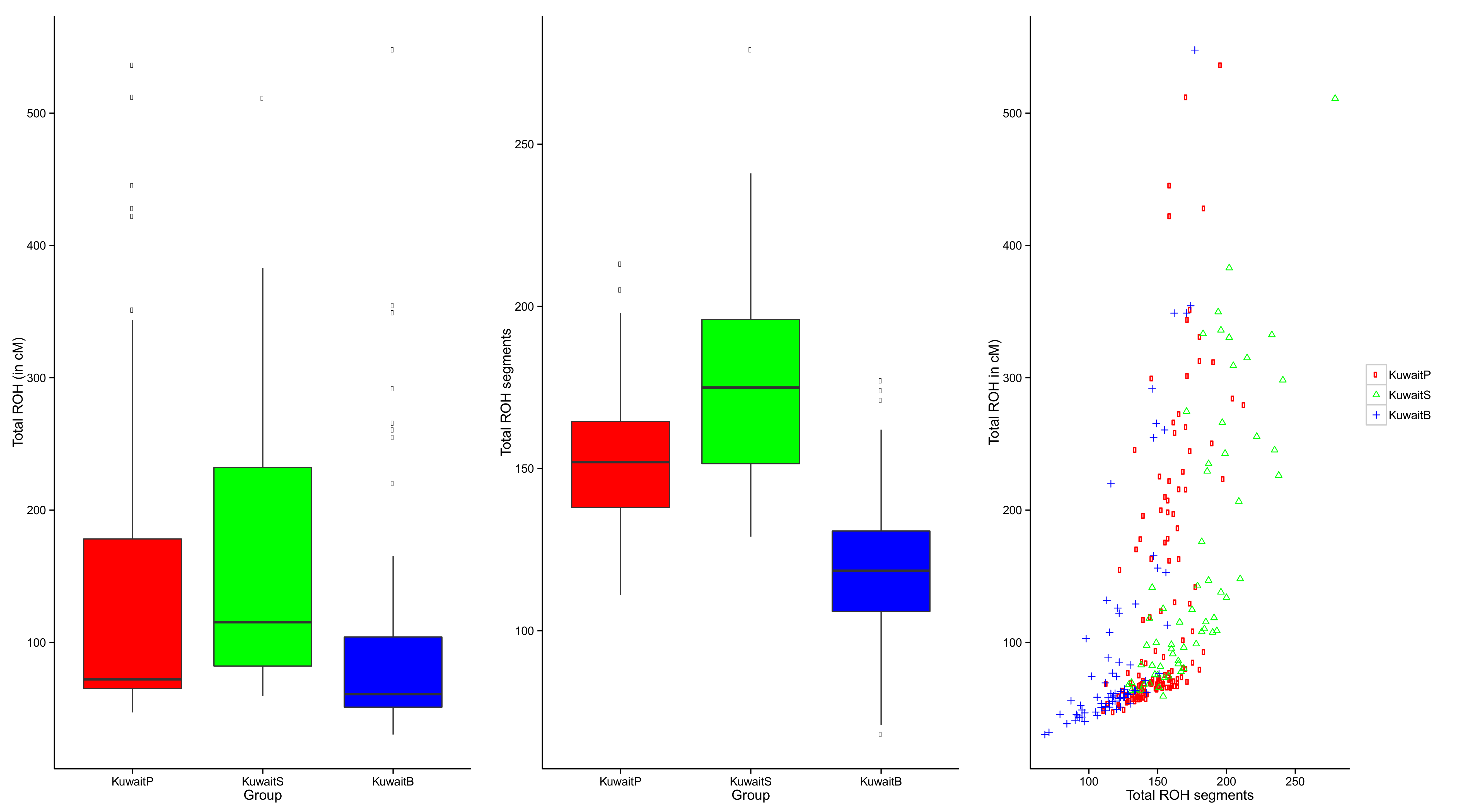

Supplement: Figure S4 — Distributions of total amount of ROH, total number of ROH segments, and total amount of ROH versus total number of ROH segments in each of the three Kuwaiti groups. (TIF) [file pone.0074913.s004.tif]

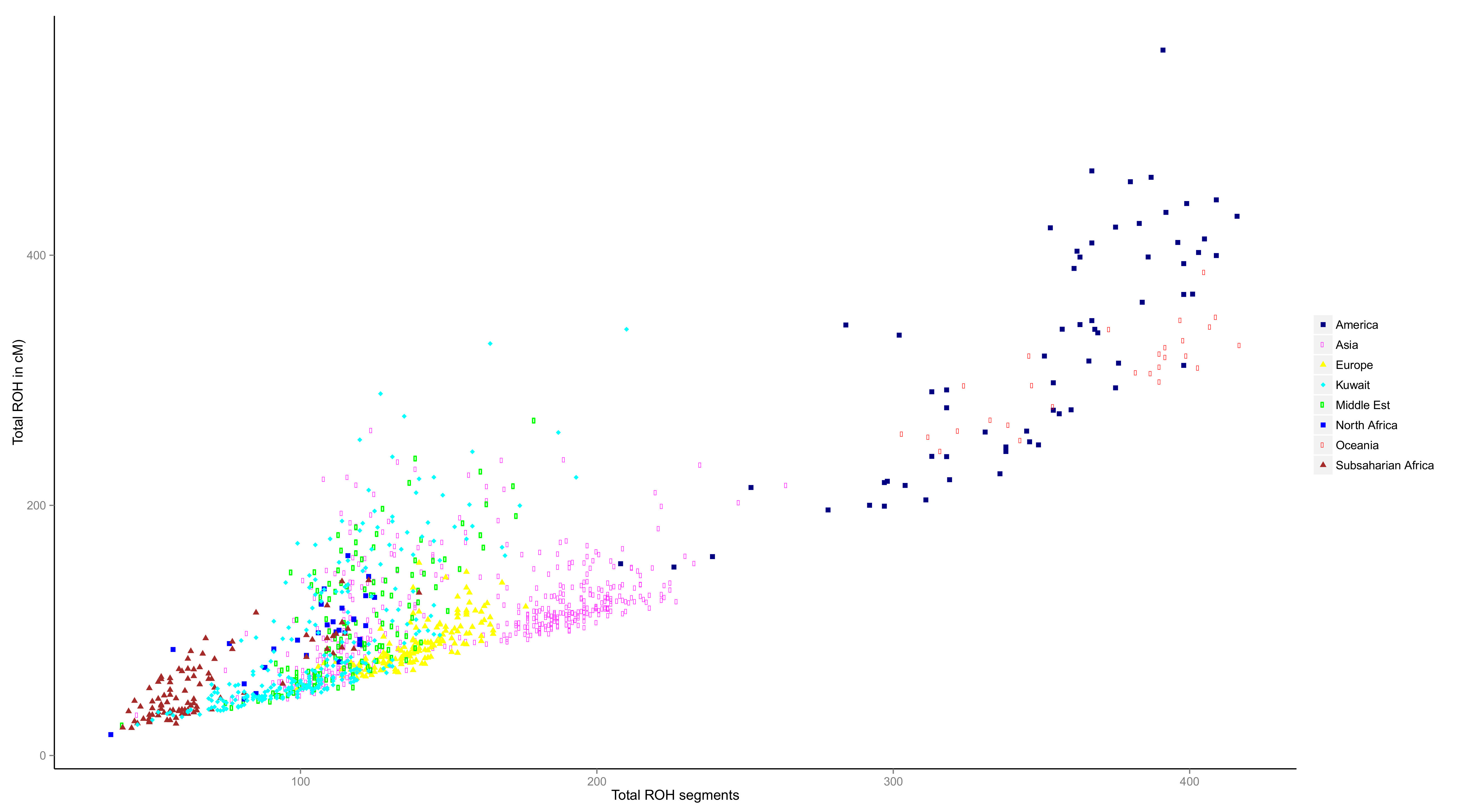

Supplement: Figure S5 — Plot of total amount of ROH versus total number of ROH segments between the Kuwaiti population and all of the HGDP populations. See Figure 4 for a representation for all of the Kuwait individuals in the background of only the representative HGDP populations. (TIF) [file pone.0074913.s005.tif]

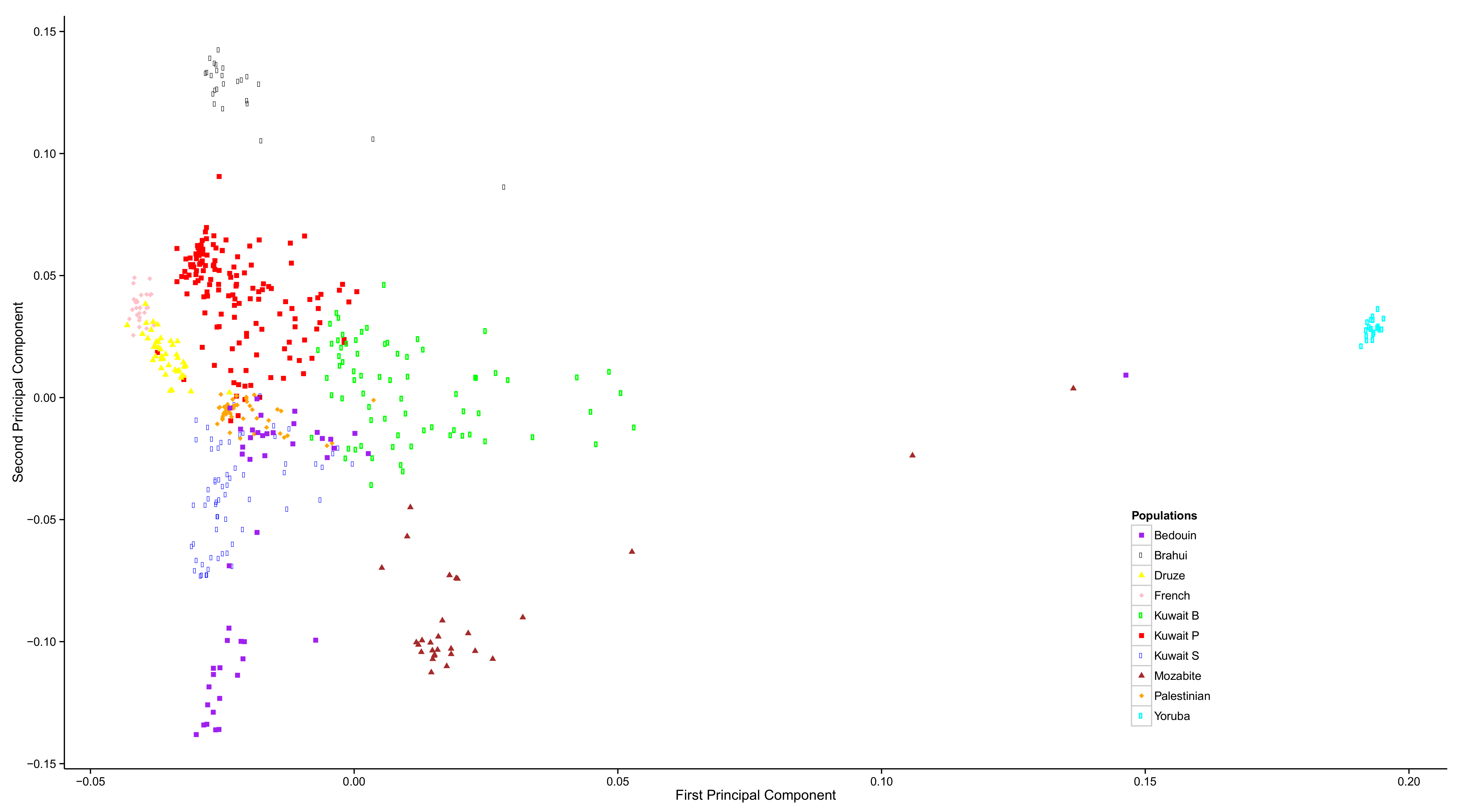

Supplement: Figure S6 — Scatter plot representing the first two principal components of merged data sets of the three Kuwaiti groups and the representative HGDP populations. The first principal component value ranges from 11.517-11.547 and the second component value ranges from 2.79-2.805 in multiple iterations of PCA. See Figure 5 for similar plot derived by excluding Yoruba population. (TIF) [file pone.0074913.s006.tif]

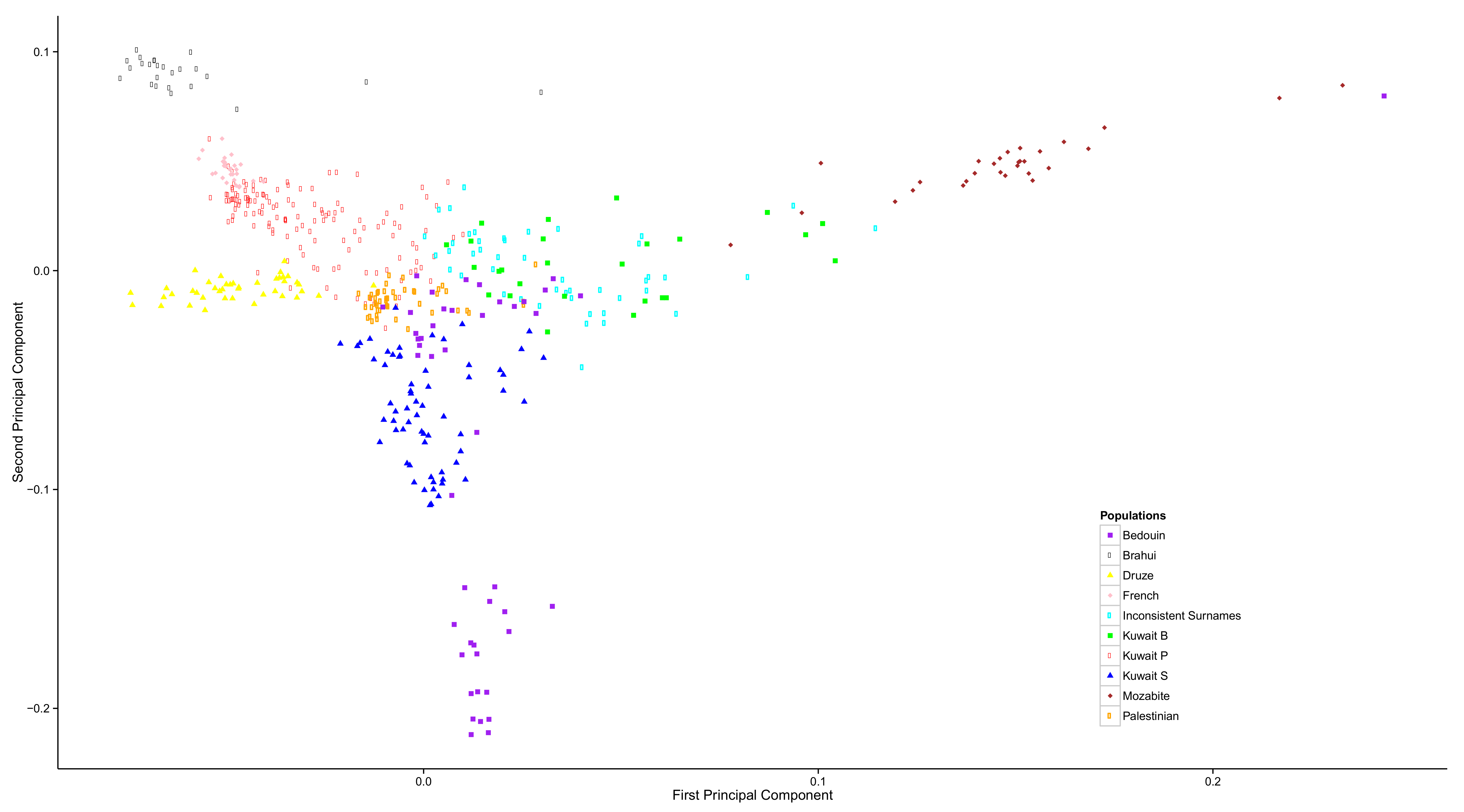

Supplement: Figure S7 — The PCA plot (namely the Figure S6) reproduced with color-coding the Bedouin and non-Bedouin surnames from the Kuwait B group. Individuals with non-Bedouin surnames, represented by Cyan color, are seen spread over the entire cluster of Kuwait B. (TIF) [file pone.0074913.s007.tif]

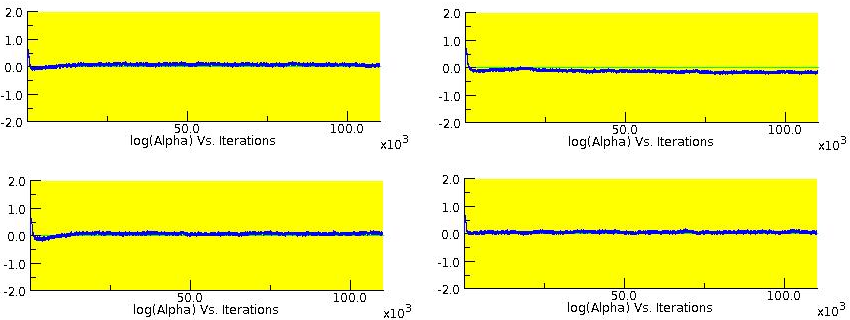

Supplement: Figure S8 — Distribution of Alpha values for 4 runs of STRUCTURE at K = 3 to identify substructure in Kuwait. (TIF) [file pone.0074913.s008.tif]
