# Supplementary material for: Genetic Substructure of Kuwaiti Population Reveals Migration History
Source: PLoS One. 2013 Sep 16;8(9):e74913. doi: 10.1371/journal.pone.0074913 (PMC3774671; doi:10.1371/journal.pone.0074913)
Supplement: Table S1 — Distribution of mean est. LnP value across multiple runs of STRUCTURE used to choose the value of K = 3. (DOCX) [file pone.0074913.s009.docx]

| **K** | **Reps** | **mean est. LnP(Data)** | **stdev est. LnP(Data)** |
| --- | --- | --- | --- |
| 1 | 4 | -9333812.15 | 13.95767 |
| 2 | 4 | -9313738.325 | 565.2319 |
| **3** | **4** | **-9299925.8** | **265.7942** |
| 4 | 4 | -9453865.175 | 231283 |
| 5 | 4 | -9934924.85 | 1133416 |
